# Revered but Poorly Understood: A Case Report of *Dendroaspis polylepis* (Black Mamba) Envenomation in Watamu, Malindi Kenya, and a Review of the Literature

**DOI:** 10.3390/tropicalmed3030104

**Published:** 2018-09-19

**Authors:** Valentine Eugene Erulu, Mitchel Otieno Okumu, Francis Okumu Ochola, Joseph Kangangi Gikunju

**Affiliations:** 1 Watamu Hospital, P.O. Box 322-80202, Watamu, Kenya; 2 Department of Pharmacy, Jaramogi Oginga Odinga Teaching and Referral Hospital, P.O. Box 849-40100, Kisumu, Kenya; mytchan88@gmail.com; 3 Department of Public Health, Pharmacology and Toxicology, Faculty of Veterinary Medicine, University of Nairobi, P.O. Box 29053-00625, Nairobi, Kenya; 4 Department of Pharmacology and Toxicology, School of Medicine, Moi University, P.O. Box 3900-30100, Eldoret, Kenya; ochola.francis@yahoo.com; 5 Department of Medical Laboratory Science, College of Health Sciences, Jomo Kenyatta University of Agriculture and Technology, P.O. Box 62000-00200, Nairobi, Kenya; jgikunju@jkuat.ac.ke

**Keywords:** black mamba, snakebite, Watamu, *Dendroaspis polylepis*, Kenya

## Abstract

The black mamba (*Dendroaspis polylepis*) ranks consistently as one of the most revered snakes in sub-Saharan Africa. It has potent neurotoxic venom, and envenomation results in rapid onset and severe clinical manifestations. This report describes the clinical course and reversal of effects of black mamba envenomation in a 13-year-old boy in the Jimba area of Malindi. The victim presented to Watamu Hospital, a low resource health facility with labored breathing, frothing at the mouth, severe ptosis and pupils non-responsive to light. His blood pressure was unrecordable, heart rate was 100 beats per minute but thready, his temperature was 35.5 °C, and oxygen saturation was 83%. Management involved suction to clear salivary secretions, several hours of mechanical ventilation via ambu-bagging, oxygen saturation monitoring, and the use of South African Vaccine Producers (SAVP) polyvalent antivenom. Subcutaneous adrenaline was used to stave off anaphylaxis. The victim went into cardiac arrest on two occasions and chest compressions lasting 3–5 min was used to complement artificial ventilation. Hemodynamic instability was corrected using IV infusion of ringers lactate and normal saline (three liters over 24 h). Adequate mechanical ventilation and the use of specific antivenom remain key in the management of black mamba envenomation.

## 1. Introduction

### Background

**Figure 1:**
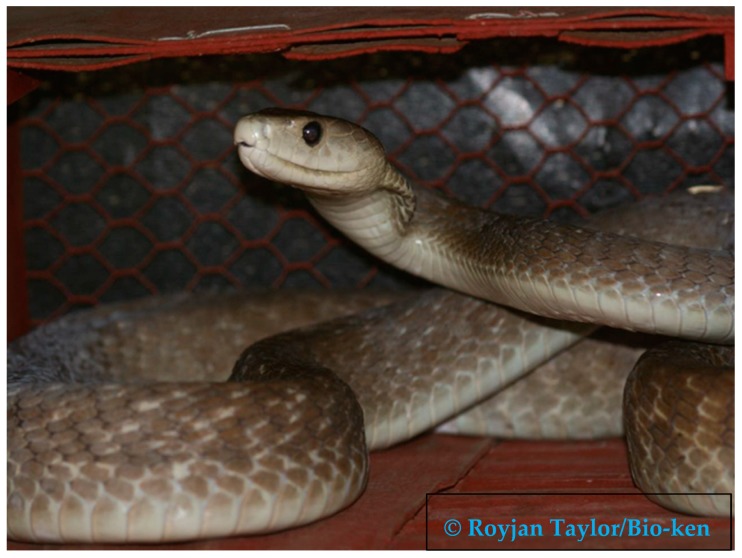
Black mamba (*Dendroaspis polylepis*).

The black mamba (*Dendroaspis polylepis*) is an olive brown- to grey-colored snake with a characteristic white belly (Figure 1).

It is native to eastern, southern and isolated parts of western Africa [1]. It is one of the species within the genus *Dendroaspis*. The others are *viridis*, *jamesoni*, and *angusticeps* [2]. The black mamba is ranked by the World Health Organization as one of the species of highest medical importance in sub-Saharan Africa. This is on account of the potency of its venom, the rapid onset and severity of clinical manifestations, and its ability to strike fast and repeatedly [3]. Nonetheless, medical literature on the threat posed by black mamba envenomation is scanty.

Few studies have evaluated the preclinical efficacy of some antivenoms against the venom of the black mamba in mouse models [4,5,6]. A 2011 study by Ochola et al., using a modification of the ‘rescue protocol’, reported that Sanofi Pasteur and Bharat Serums antivenom were effective in mitigating experimental black mamba envenomation in mice [6]. The study also reported symptoms of black mamba envenomation in a mouse model [6]. The rescue protocol involves simulating actual snakebite scenarios. In the protocol, mice are injected with a lethal dose of venom and a time interval is allowed for symptoms of envenomation to manifest. Test antivenoms are then administered and the efficacy of the antivenom is evaluated based on the quantity (in milligrams) of venom neutralized by the antivenom. Two other studies [4,5] adopted the World Health Organization (WHO) ‘pre-mix’ strategy where venom and antivenom are first pre-mixed, then incubated and the mixture is administered in mice. Among these authors, Laustsen and colleagues reported that polyvalent antivenoms manufactured by South African Vaccine Producers (SAVP) and VINS Bio products were effective against black mamba envenomation in mice [4]. On their part, Harrison and colleagues [5] suggested that antivenoms manufactured by PAN AFRICA, INOSAN Biopharma, and Sanofi Pasteur were not effective against all the medically important snake species in sub-Saharan Africa. In our opinion, it is damning that, for a snake that is native to Africa, recent medical literature on black mamba envenomation in humans is largely from Europe [7,8,9].

However, this is not without reason. There is a total lack of emphasis on venomous snakebite management in medical schools in sub-Saharan Africa. Additionally, there are few poison control centers and no snakebite management protocols in the region. Clinicians may, therefore, be ill-equipped to effectively manage venomous snakebites, including those of the black mamba. We present a case report of the clinical course of black mamba envenomation in a rural area of Kenya and the medical steps undertaken to reverse the effects of envenomation in a low-resource health facility.

## 2. Case Presentation

**Figure 2:**
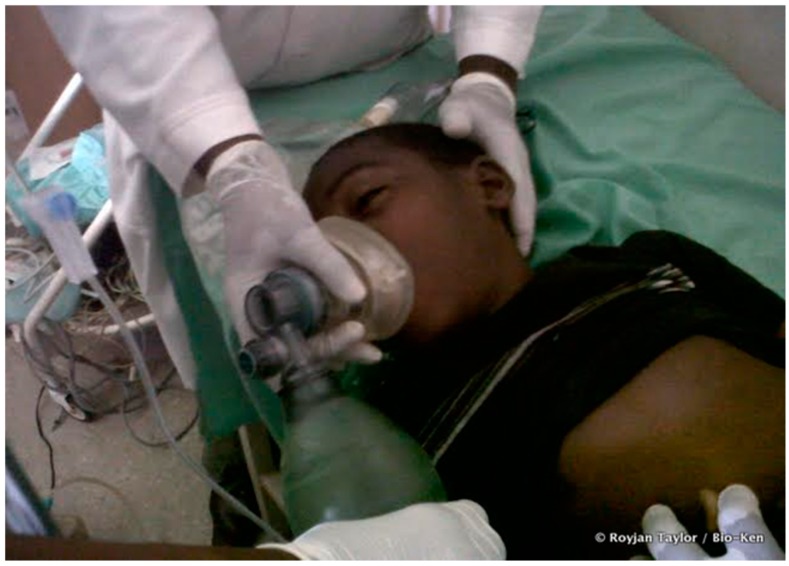
Victim of the black mamba bite.

A 13-year-old boy from Jimba in Watamu, Malindi Kenya was playing with his friends in a bushy area surrounding their homestead. Suddenly, he was bitten on his right leg by a snake which was described by onlookers as long and brown in colour. Upon noticing the bite, he rushed home where a black stone was tied at the site of the bite and prayers were invoked. Soon after, he started frothing at the mouth and had labored breathing. A cousin rushed him to the nearest health facility (Gede dispensary in Watamu), where they were immediately referred to Bio-Ken Snake Farm. They arrived at Bio-Ken Snake Farm about an hour after the bite had occurred. Once at Bio-Ken, he was immediately driven by Sanda Ashe, a director at Bio-Ken to Watamu Hospital, a journey which took less than 10 min. On arrival, two bite marks could be seen on his right mid-shin with no signs of edema. He was sweating profusely, hypersalivating with severe ptosis, and his pupils were non-responsive to light (Figure 2). His blood pressure was unrecordable, his heart rate was 100 beats per minute but thready, and body temperature was 35.5 °C. He was semi-comatose (Glasgow Coma Scale: 9/15) and oxygen saturation was 83%. Evaluation of the respiratory rate and ECG were not performed.

**Figure 3:**
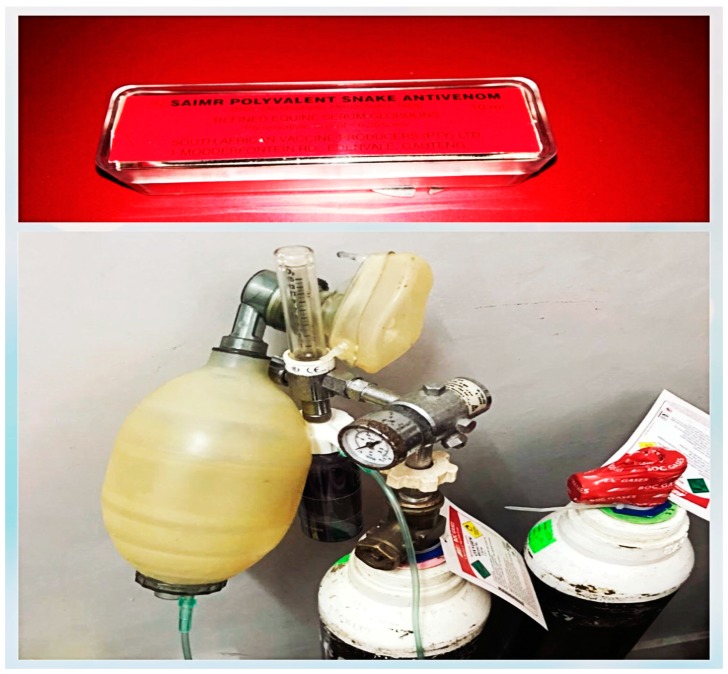
Antivenom used on the victim and the positive pressure oxygen delivery system used in the resuscitation.

Suction was initiated to clear the secretions and oxygen administered via an ambu-bag. Two ampoules (20 mL) of South African Vaccine Producers (SAVP) polyvalent antivenom was administered to the patient by rapid IV push. Furthermore, two other ampoules (20 mL) were given in a 500 mL IV infusion of 0.9% *w*/*v* normal saline. Extra IV lines were run at 10, 15, and 20 min intervals. In total, four vials of antivenom were used. About 10 min after antivenom infusion, the patient developed severe urticaria for which adrenaline 0.5 mL was administered subcutaneously. Ambu-bagging was the only means of resuscitation available and the victim was bag ventilated over a period of two hours with oxygen via an oxygen cylinder (Figure 3).

**Figure 4:**
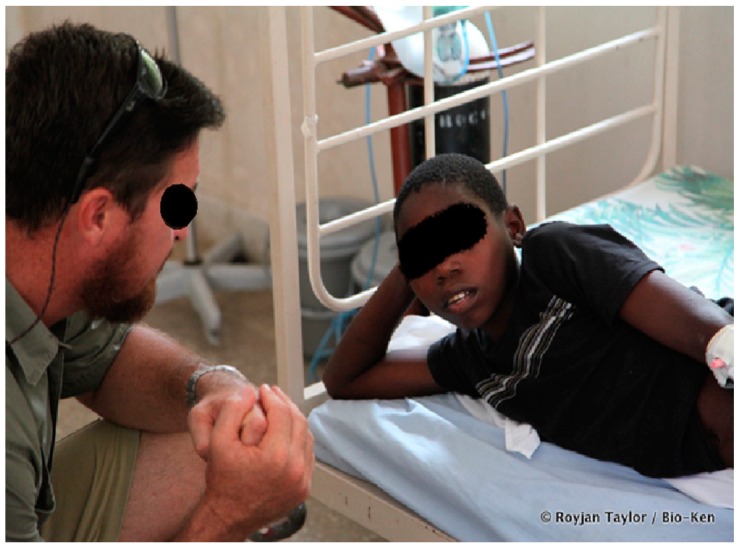
Victim of the black mamba bite (on the right) after reversal of envenomation.

A clinical officer, a nurse, a nurse assistant, and the physician all took turns hand-ventilating the patient. In the course of ambu-bagging and IV infusion of antivenom, the victim went into cardiac arrest on two occasions. There were no cardiac sounds on auscultation and his pulse could not be detected. Similarly, no readings could be observed on the screen of the pulse oximeter. Chest compressions lasting 3–5 min were then immediately initiated on both occasions to complement artificial ventilation. This was stopped when cardiac sounds could be heard on auscultation, and readings could be observed on the screen of the pulse oximeter. At this point, his pulse was fluctuating and, thus, presumed post-resuscitation hemodynamic instability was corrected using IV infusion of Ringers lactate and normal saline (three litres over 24 h) until his pulse stabilized at 95 BPM. Ambu-bagging was continued for a further five and a half hours until pulse oximeter readings stabilized at 95–98% and a strong pulse could be felt. At this time, his temperature was 36.5 °C, BP was 110/70, pulse was 88/min (and strong) and oxygen saturation was 99%. He was then weaned off oxygen and admitted for further monitoring. Over the course of the next day, he could breathe on his own and had no neurological impairment. (Figure 4). He could recall all the events leading up to his arrival at the hospital and was also able to identify members of his family who had visited him at the hospital. Bio-Ken later confirmed the snake to be a black mamba. Given the remarkable progress made by the patient, the absence of neurological impairment or any other organ dysfunction, the decision was made not to refer the patient.

It is worth noting that Watamu Hospital is a small private hospital with a capacity of eight beds. The facility is located 422 km southeast of Nairobi (3.3622° S, 40.0017° E) in a rural area on Turtle Bay Road in Watamu. The hospital has a basic laboratory and the bite occurred on a day (Sunday) when the hospital operates with minimum staff. Moreover, the nearest referral hospital (Malindi County Hospital) is 25 km away and lacks appropriate antivenom. Therefore, the main focus of this intervention was resuscitation of the patient. Apart from what we have highlighted, it was not possible to do more. Thankfully, the patient had no neurological impairment or any organ dysfunction. He is currently volunteering at the Bio-Ken Snake Farm where he assists in the care of the captured snakes.

## 3. Discussion

The black mamba has a wide geographical distribution in sub-Saharan Africa and bites from the snake often result in high levels of morbidity, disability, or mortality [4]. Ironically, most of the recent reports of black mamba bites in medical literature have emanated from outside the continent, mainly involving snake breeders or handlers of exotic snakes. Between 2011 and 2017 there have been reports of black mamba bites in Switzerland, Germany, and the Czech Republic [7,8,9]. Within the continent of Africa, Strover [10], Saunders [11], Blaylock [12], Crisp [13], Hilligan [14], Naidoo [15] and their colleagues have reported on black mamba bites in Zimbabwe (1967, 1980, 1982), South Africa (1986, 1987), and Swaziland (1987). To the best of our knowledge, this is the first documented case report of a confirmed black mamba envenomation in East Africa that was successfully treated.

Black mamba venom has rapid onset of action and symptoms have been reported to occur anywhere between 10 and 15 min post-envenomation [16,17]. The clinical presentation may vary markedly owing to the array of toxic proteins that make up the venom. Initially, envenomation may be characterized by little or no swelling or bleeding [1,8,10,15], nausea [17], and conjunctival congestion [11,17]. A few minutes later, the victim may begin to sweat profusely [8,17], hypersalivate [10], vomit [15,17], complain of a strange taste in the mouth, and tingling sensations throughout the body leading to paresthesia and weakness [8,17]. Thereafter, the victim may begin to appear as if he/she has just been immersed in water (‘gooseflesh’) [17] and may exhibit labored breathing [8,10,15]. Then, ptosis [17], paralysis of vocal cords, muscles of the pharynx and deglutition [10,15] as well as general flaccid paralysis may be observed. Thus, victims may be conscious but unable to speak. Several hours after envenomation, victims may become restless and may violently thrash about with arms and legs [10,15]. Eventually, generalized fasciculations may set in [8,15] and cardio-respiratory collapse may lead to death [18]. Clinically, tachycardia, hypotension, polyuria, and a high leucocyte count may be observed. There may also be sinus bradycardia, glycosuria, fall in Hb and creatinine levels [8,10,15].

Toxic proteins in black mamba venom are particularly neurotoxic. Proteomic characterization of the venom has revealed 41 different proteins and one nucleoside [4]. Major proteins reported include dendrotoxins, α-neurotoxins, muscarinic toxins, fasciculins, calciseptine, mamba intestinal toxins, and mambalgin [4,19,20,21,22,23]. Minor proteins include metalloproteinases, hyaluronidase, prokinecitin, nerve growth factor, phospholipase A_2_, 5’-nucleotidase, and phosphodiesterase [4].

Dendrotoxins primarily act on the voltage-dependent potassium channels where they potentiate effects of acetylcholine by facilitating its release at pre-synaptic nerve terminals, thereby eliciting excitatory effects [11,24,25]. α-Neurotoxins bind to the nicotinic cholinergic receptors at the motor end-plates of muscle fibers and have the capacity to cause flaccid paralysis that leads to labored breathing and death [19]. Muscarinic toxins bind to muscarinic cholinergic receptors [26], and fasciculins inhibit anticholinesterase; thus, there is an increase in the levels of acetylcholine at neuromuscular junctions, which manifests as generalized, long-lasting fasciculations [20]. Calciseptine selectively inhibits L-type calcium channels, smooth muscle contraction, and cardiac function [20], while mamba intestinal toxins and mambalgin block the acid-sensing channels associated with pain [21,22].

In the case of a black mamba bite, first aid should aim to inhibit the systemic absorption of venom and prevent effects that may be life-threatening by immediately evacuating the patient to a medical facility [27]. Making incisions, sucking the venom from the wound, tying tourniquets/arterial bands around the limb, local application of ice packs, snake stones, or herbal medicine are contraindicated [27]. Electric shocks and washing the wound are construed to interfere with the wound and may pre-dispose the wound to infection, increased bleeding or increase the rate of absorption of the venom [27]. Instead, re-assurance of the victim, pressure immobilization of the affected limb—either by bandage or cloth to hold the splint—and prompt evacuation of the victim to a medical facility are recommended [16,27].

Delay in seeking treatment for a black mamba bite often results in a poor prognosis and even death [17]. There may be instances when a black mamba bite could be a ‘dry bite’. However, the absence of fang marks should not be used as criteria to rule out envenomation. In the same vein, the presence of fang marks should not be used as a confirmation of envenomation [7]. The recommended initial dose of SAIMR polyvalent antivenom (South African Vaccine Producers) is 2 vials (20 mL). However, additional antivenom may be titrated upwards against the clinical syndrome of black mamba envenomation [28]. Intravenous saline drips may be used to counteract excessive sweating [10] and suction may be useful in clearing secretions [15]. Fluid infusions may be important in stabilizing blood pressure [15] and sinus bradycardia has been shown to respond to intravenous atropine [15]. Blood pressure may also be stabilized by volumosubstitution and temporarily administering a low dose of norepinephrine [8].

In the event of pulmonary edema, administration of furosemide and mannitol with subsequent administration of hydrocortisone and prophylactic antitetanus injection has been shown to be beneficial [8]. Intubation and mechanical ventilation should be initiated with analgosedation [8]. Additionally, it may be necessary to transfer the patient to the intensive care unit (ICU) under continued sedation, ventilation, and intermittent muscle relaxation [8]. Tracheostomy may be a necessity in instances where victims have been unable to be weaned off the ventilator [15]. Continuous monitoring of the victim is needed before extubation can be considered [15].

To address fasciculations, increased carbon dioxide saturation and hyperthermia, sedation with diazepam, midazolam, thiopental, and magnesium sulphate have been shown to be beneficial [8]. Fasciculations may be made to recede further with the use of a bolus 0.5 mg/kg dose of atracurium [8]. Often, venom components or the immunoglobulins present in the antivenoms may cause anaphylaxis [7]. Nonetheless, anaphylaxis should not be a hindrance to antivenom use [7,11]. Severe anaphylaxis may warrant temporary discontinuation of the antivenom infusion and subsequent stabilization of the patient before resumption of infusion at a slower rate [7]. For patients at a high risk of allergic reactions, 1 mL of antivenom may be diluted with 9 mL of normal saline and infused to the patient and observations made [7].

In our case, tragedy was averted largely because of five major reasons: (1) The on-hand availability of equipment for mechanical ventilation and cardiac pulmonary resuscitation; (2) The great presence of mind of the victim’s cousin in rushing him to hospital despite the insurmountable odds; (3) The effectiveness and swift use of the SAIMR polyvalent antiserum; (4) The capacity of subcutaneous adrenaline to stave off anaphylaxis; and (5) rapid IV access. Our report highlights the importance of swift and evidence-based medical intervention in the event of black mamba envenomation. Mechanical ventilation and the use of effective antivenom remain key in managing black mamba envenomation. Clinicians manning casualty departments of hospitals in sub-Saharan Africa should be conversant with the pathophysiology and clinical toxicology of venomous snakebites and the appropriate measures to be instituted. Moreover, there is a need to lay more emphasis on snakebite management in the curriculum of medical schools in Africa.
